# Insulin sensitivity is associated with the observed variation of de novo lipid synthesis and body composition in finishing pigs

**DOI:** 10.1038/s41598-022-18799-0

**Published:** 2022-08-26

**Authors:** Hector Hernando Salgado, Candido Pomar, Marie-France Palin, Hélène Lapierre, Marie-Pierre Létourneau-Montminy, John P. Cant, Aline Remus

**Affiliations:** 1 Sherbrooke Research and Development Centre, Agriculture and Agri-Food Canada, Sherbrooke, Canada; 2 Department of Animal Science, Université Laval, Québec, Canada; 3 Department of Animal and Poultry Science, University of Guelph, Guelph, Canada

**Keywords:** Fat metabolism, Insulin signalling

## Abstract

Variations in body composition among pigs can be associated with insulin sensitivity given the insulin anabolic effect. The study objectives were to characterize this association and to compare de novo lipogenesis and the gene expression in the adipose tissue of pigs of the same genetic background. Thirty 30–95 kg of body weight (BW) pigs, catheterized in the jugular vein participated into an oral glucose tolerance test (OGTT; 1.75 g glucose/kg of BW) to calculate insulin-related indexes. The 8 fattest and the 8 leanest pigs were used to determine the relative mRNA abundance of studied genes. The rate of lipogenesis was assessed by incorporation of [U-^13^C]glucose into lipids. The QUICKI and Matsuda indexes negatively correlated with total body lipids (r =  − 0.67 and r =  − 0.59; *P* < 0.01) and de novo lipogenesis (r =  − 0.58; *P* < 0.01). Fat pigs had a higher expression level of lipogenic enzymes (*ACACA*, *ACLY*; *P* < 0.05) than lean pigs. The reduced insulin sensitivity in fat pigs was associated with a higher expression level of glucose-6-phosphate dehydrogenase (*G6PD*) and a lower expression of peroxisome proliferator-activated receptor-gamma (*PPAR-γ*). In conclusion, pigs with increased body lipids have lower insulin sensitivity which is associated with increased de novo lipogenesis.

## Introduction

Lipid deposition in pigs is affected by many factors, such as nutrition, sex, breeds, environment and others^1^. However, when these factors are considered an important variation in body composition among pigs is still observed^2,3^, indicating that pigs respond differently to the same amount of ingested nutrients, for example, amino acids^4,5^. Understanding the factors associated with this variation can help producers to improve nutrient utilization and to manipulate body composition in pigs. Differences in nutrient utilization and subsequent variations in body lipids among pigs might be partly explained by individual variations in metabolic processes involved in energy regulation. Among them, insulin sensitivity is a key candidate given that insulin is a positive regulator of fatty acid synthesis and body adiposity^6,7^. Indeed, when insulin was infused into growing pigs, while maintaining blood glucose concentrations, the incorporation of glucose into fatty acids increased by 51%^8^.

The main site for de novo lipogenesis in pigs is the adipose tissue with glucose as the preferred precursor^9^. In addition to its role in the synthesis and storage of lipids, the fat cells produce and secrete hormones called adipokines, which modulate insulin sensitivity and can influence energy metabolism and expenditure^10^. Lipid dysregulation in *db*/*db* and *ob*/*ob* obese mouse models^11^, and high body fat in insulin resistant humans^12^ are examples of the strong link between insulin sensitivity and intermediary metabolism in adipose tissue. Although other hormones such as leptin are also implicated in adiposity regulation, insulin is a prime candidate in signalling adiposity because it better maps acute changes in energy metabolism and lipogenesis, and because plasma insulin follows the dynamic between the energy ingested and how it is retained in the body (e.g. as lipids or protein) over time^6^. Unlike plasma insulin, plasma leptin levels take longer to respond to changes in energy dynamics^13,14^.

Some studies have reported greater postprandial serum levels of insulin in obese pig lines than in lean lines^15,16^, and others have reported different insulin sensitivities between these lines^17^. For example, Iberian pigs, which are considered an obese genetic line had lower whole-body insulin sensitivity but increased β-cell function compared with lean Landrace pigs^17^. Although insulin sensitivity has been considered when comparing insulin responses between breeds, little is known about the relationship of insulin sensitivity with the observed variations in body composition within breeds or animals from the same genetic background. Therefore, the objectives of this study were to characterize the association between body composition, lipid synthesis and whole body insulin sensitivity in finishing pigs of the same genetic background, and to evaluate whether fat and lean pigs differ in the expression of genes in adipose tissue involved in lipid metabolism and insulin sensitivity, which could potentially result in different rates of de novo lipogenesis.

## Materials and methods

The animals were cared for according to a recommended code of practice^18^ and the guidelines of the Canadian Council on Animal Care^19^. The animal trial was approved by the Ethical and Animal Welfare Committee of the Sherbrooke Research and Development Centre, Agriculture and Agri-Food Canada (Sherbrooke, QC, Canada). This study was carried out in compliance with the ARRIVE guidelines for the reporting of animal experiments.

Thirty barrows (36 ± 3.8 kg of body weight (BW)) of the same genetic background (Yorkshire-Landrace × Duroc; Benjoporc et Akama, Sainte-Geneviève-de-Batiscan, QC, Canada) were shipped to the Agriculture and Agri-Food Canada swine complex (Sherbrooke, QC, Canada). Pigs were allocated to one pen with a concrete floor in the same mechanically ventilated room. Pigs were fed ad libitum with a commercial diet and had free access to water before and during the experimental period. Room temperature was adjusted from 22 °C at the pig’s arrival to 18 °C at the end of the experiment. Fluorescent lighting was controlled by a timer and ensured 12 h of light daily. The experiment included two trials. The first one consisted of an oral glucose tolerance test (OGTT) to evaluate the variability among animals of plasma glucose, insulin response and insulin sensitivity to the same dose of oral glucose and their relationship with body lipids content. The second trial was performed to determine de novo lipogenesis rate using a bolus of [U-^13^C]glucose, and the relative mRNA abundance of lipogenic enzymes and of markers associated with insulin sensitivity in fat and lean pigs.

### Trial 1

Upon reaching 95 kg BW, pigs were placed in individual cages for one week. During this period, a commercial diet (2.7 kg/day; Table 1) in the form of three equal meals was offered. Two days before the OGTT, pigs were fitted with a catheter in the jugular vein following a non-surgical procedure^20^. On test day (day 7), a 1.75-g/kg of BW of glucose, mixed with a 300 ml solution of flavour-free gelatin (hydrolyzed collagen) and water (23.3 mg/ml of water), was orally offered (0800) to each pig after 18 h of fasting^21^. The use of gelatin allows precise delivery of the desired amount of glucose by mimicking a meal, without stressing the animals^22,23^. Blood samples were collected at − 20, − 10 and 5, 10, 15, 20, 25, 30, 45, 60, 90, 120, 150, 180, 210, 240, 300 and 360 min after the ingestion of glucose. For each collection time, a 10-ml blood sample was collected for insulin and C-peptide analyses (EDTA tubes) and a 2-ml sample for glucose analysis (potassium oxalate monohydrate/sodium fluoride tubes). Blood samples were stored on ice until centrifugation for 12 min at 1800×*g* at 4 °C. The collected plasma was kept at − 20 °C until determination of insulin, glucose and C-peptide concentrations. At the end of the first trial, the pigs were maintained in single pens and fed ad libitum until the beginning of the second trial.

**Table 1:** Chemical composition of the commercial diet used during trials 1 and 2.

Composition	% of DM
Dry matter	87.2
CP	14.8
Lipids	5.2
Starch	53.2
Amylose, % starch	24.7
Amylopectin, % starch	75.4
NDF	7.6
ADF	3.2

*DM* dry matter, *CP* crude protein, *NDF* neutral detergent fiber, *ADF* acid detergent fiber.

#### Biochemical analyses and calculations

Plasma glucose was measured by an enzymatic calorimetric assay (No 997-03001, Wakolife Sciences, Mountain View, CA, USA). Insulin concentration was determined with a porcine insulin commercial RIA kit (#PI-12K; EMD Millipore Corporation, Saint Charles, MO, USA) and C-peptide concentration was measured with an ELISA kit (C-peptide porcine No 10-1256-01, Mercodia Inc, Winston-Salem, NC, USA). Plasma insulin, C-peptide and glucose responses were evaluated by computing the total area under the curve (AUC_0–360_) using the trapezoidal method between 0 and 360 min post-glucose ingestion. Indexes to assess insulin sensitivity in humans [QUICKI and Matsuda index (MI)] were calculated during the basal and the post-glucose ingestion period. Delta glucose and delta insulin values were calculated by determining the difference between the maximum glucose or insulin concentrations after ingestion of glucose and their respective basal concentrations.

Insulin sensitivity in the basal state was estimated using the quantitative insulin sensitivity check index (QUICKI), which was calculated as proposed by Katz, et al.^24^ and as follows:

$$QUICKI =\frac{1}{\left[\log\left(fasting \,plasma \,insulin\right)+\log\left(fasting \,plasma \,glucose\right)\right]}.$$

The Homeostasis model assessment (HOMA) was used to estimate insulin resistance (HOMA-IR) and β-cell function (HOMA-%B) at basal conditions as follows (https://www.dtu.ox.ac.uk/homacalculator/):

$$HOMA-IR=\frac{\left(fasting\, plasma \,glucose \times fasting\, plasma\, insulin\right)}{22.5},$$

$$HOMA-\%B=\frac{20\, \times\, fasting\, plasma\, insulin}{fasting\, plasma \,glucose-3.5}.$$

The HOMA model assumes that individuals with no insulin resistance have 100% β-cell function and an insulin resistance (HOMA-IR) of 1.

The Matsuda index (MI) was also used to estimate whole-body insulin sensitivity with insulin and glucose concentrations measured during the OGTT^25^ as follows:

$$MI = \frac{\mathrm{10,000}}{\sqrt{\left(fasting\, glucose \,\times \,fasting\, insulin\right)\times (glucose\, OGTT\, mean \,\times\, insulin\, OGTT\, mean}}.$$

The C-peptide: insulin ratio was used as an indicator of hepatic insulin extraction and clearance^26^. Accute pancreatic insulin secretion during the OGTT was assessed with the oral disposition index (oDI_cpep_) using the C-peptide and glucose measurements during the OGTT^27^, as follows:

$$oDIcpep=\frac{{\Delta\, AUC-C-peptide}_{0-30 \,{\text{min}}}}{{\Delta \,AUC-glucose}_{0-30\,{\text{min}}}}\times MI.$$

A lower oDI_cpep_ indicates a reduced ability for β-cells to secrete insulin to match the level of whole-body insulin sensitivity. In addition, the β-cell function from the OGTT was calculated as the ratio of the area under the curve of C-peptide from 0 to 120 min to the area under the curve of glucose from 0 to 120 min as follows^28^:

$$C-peptide/glucose=\frac{{\Delta \,AUC-C-peptide}_{0-120 \,{\text{min}}}}{{\Delta \,AUC-glucose}_{0-120 \,{\text{min}}}}.$$

#### Body composition

Total body fat and lean content were measured by dual-energy X-ray absorptiometry (DXA) 5 days before the OGTT using a densitometry device (GE Lunar Prodigy Advance; GE Healthcare, Madison, WI, USA). Pigs were scanned in the prone position using the total body scanning mode (GE Lunar enCORE, version 8.10.027; GE Healthcare). Anaesthesia was induced with sevoflurane (7%) and maintained with isoflurane (5%) during the scans. The DXA total body lean, and fat mass value were converted to their protein and lipid whole-body chemical equivalents^29–31^.

### Trial 2

One week after the end of trial 1, the 8 fattest and the 8 leanest pigs from the original group of 30 were selected to participate in the second trial (108.9 ± 2.8 kg of BW). As in trial 1, pigs were kept in individual cages for 1 week before determination of lipogenesis rates and sample collection. Pigs were fed with the same commercial diet (2.9 kg/day; Table 1) once per day, except for the last 4 days of the study (including the day of the bolus injection), when pigs were fed 6 times per day every 4 h. This was done to achieve a relatively steady state of nutrient absorption and utilization, which is simplifies determination of de novo lipogenesis^32^ by isotope dilution. Two days before the test, the pigs were catheterized a second time in the jugular vein following the same procedure as in tri0al 1. Two biopsies of subcutaneous (sc) adipose tissue (106 mg) were taken on day 1 after 18 h of fasting to determine the natural abundance of ^13^C in lipids and the basal gene expression levels. Biopsies were taken under local anaesthesia (EMLA, lidocaine 2.5% and prilocaine 2.5%) using a standard biopsy punch of 8 mm (REF 33–37, Manufacturer Miltex, Inc. York, PA 17402 USA) in the midline of the right side of the pig’s back between 4 and 12 cm distal to the last lumbar vertebra. The biopsy punch allowed the sampling of the sc fat layer immediately under the skin (outer fat layer) and, in some samples, the middle fat layer was also visible^33^. The skin and the middle fat layer were cut and discarded, and the outer fat layer sample was kept at − 80 °C.

The detailed experimental procedure for determination of de novo lipogenesis is described in Salgado et al.^34^. Briefly, on the last day of the trial, an intravenous bolus injection of [U-^13^C]glucose (99% enriched,12 mg/kg BW; 1.6 mmol/g of saline) was administered via the catheter to each pig 2 h after the morning meal. The injection lasted 1 min, and time 0 was considered to be the beginning of the injection. Blood samples were collected − 5, 2, 4, 6, 9, 12, 15, 20, 30, 40, 60, 80, 100, 120, 150, 180, 210, and 240 min after the labelled glucose injection, to analyze plasma glucose isotopic enrichment (IE) and concentration (heparinized tubes). Immediately after sampling, blood was placed on ice before centrifugation (15 min, 1800×*g* at 4 °C). Plasma was stored at − 80 °C for the determination of plasma glucose IE and at − 20 °C for concentration analyses. Four hours after the bolus injection, the pigs were euthanized using a penetrating captive bolt gun followed by exsanguination, and sc adipose tissue (outer layer) was immediately collected from the same site as the biopsies but in the left side of the pig. All adipose tissue samples were frozen in liquid N and stored at − 80 °C until analyzed for ^13^C IE and gene expression.

#### Isotopic enrichment of plasma glucose and of lipids from adipose tissue

Samples of plasma were deproteinized with a 2:1 mixture of acetonitrile and ethanol, and derivatized with acetic anhydride. The ions 242 and 247 were quantified by gas chromatography-mass spectrometry (GCMS:GC 6890N network GC system coupled to MS 5973 Network; Agilent Technologies, Wilmington, DE, USA) in electron impact mode. The GCMS analyzes the whole derivatized molecule of glucose, thus yielding the results as mole percent excess (MPE). Lipid extraction from adipose tissue was performed with slight modifications of the technique by Shahidi^35^. Briefly, 80 mg of adipose tissue was homogenized in 3 ml of a methanol-chloroform (2:1) solution and 0.2 ml of water for 30 s. Then, 2 ml of a chloroform-water (1:1) solution was added to the sample and centrifuged at 3300×*g* for 15 min at 10 °C. After evaporating the solvents under N_2_, the extract was dried at 55 °C for 2 h. The ^13^C IE and C contents of the extracted lipids were determined after combustion on an elemental analyzer interfaced to an isotope ratio mass spectrometer (IRMS; Delta Advantage, Thermo, Germany). The results are expressed as atom percent excess (APE).

#### Lipogenesis

De novo lipogenesis was determined by following the incorporation of ^13^C from the labelled glucose into lipids according to the procedure proposed by Salgado et al.^34^. Assuming that all the increase of the IE lipids originated from the labelled glucose, the R_glucose-lipids_ was calculated as follows:

$$\mathrm{R}glucose-lipids\left(t\right)=\frac{\Delta\, IE \,lipid\, \times\, Cm\, extracted\, lipids}{\sum_{0}^{t}IE \,plasma\, glucose\, \times\, Cm\, glucose},$$

where the R_glucose-lipids_ [(µg glucose)/(min × g of lipids)] is determined from time 0 to time t. The Δ IE_lipid_ is the difference between the IE of lipids at time t (slaughter) and natural abundance in APE. The Δ IE_plasma glucose_ is the cumulative area under the curve of the plasma glucose IE (MPE) above natural abundance over time, determined by the integration of the double-exponential curve IE_glucose_(t) = α e^(–*k1*t)^ + β e^(–*k2*t)^. The Cm_extracted lipids_ represent the relative contribution of the C mass to the total mass of lipids in each adipose tissue sample, which was obtained from the elemental analyzer. The Cm_glucose_ was set to 0.40, equal to the ratio of the C mass to total mass (6 × 12/180) where 6 is the number of C in 1 mol of glucose, 12 is the C-atomic mass (g) and 180 is the glucose molar mass (g).

#### Relative mRNA abundance of studied genes in the adipose tissue

Adipose tissue samples (~ 100 mg) were homogenized in 1 ml of TRIzol reagent (Invitrogen Life Technology, Burlington, ON, Canada), incubated at room temperature for 5 min and 200 µl of CHCL_3_ was then added to the solution to remove lipids. After centrifugation at 12,000×*g* for 5 min (at 4 °C), the aqueous phase was transferred in a new tube and an equal volume of 70% ethanol was added. This solution is then used for total RNA isolation using the RNeasy Lipid Tissue Mini Kit (Qiagen, Toronto, ON, Canada), which included a DNAse I digestion step. Extracted RNA concentration and integrity were assessed with the NanoDrop 1000 Spectrophotometer (Thermo Fisher Scientific, Wilmington, DE, USA) and by nucleic acid electrophoresis with the Agilent 2100 Bioanalyzer instrument (Agilent, Santa Clara, CA, USA). Reverse transcription of total RNA (1 µg) to cDNA was performed with the Superscript IV Reverse Transcriptase (200 U/ml; Thermo Fisher Scientific) using oligo(dT) 20 primers.

The relative mRNA abundance of genes known to be involved in lipogenesis and insulin sensitivity was quantified using real-time qPCR analyses. Table 2 provides the complete list of selected genes with their corresponding GenBank accession numbers. Amplifications were performed in a 10-µl reaction volume containing 5 ml of 2× Power SYBR™ Green PCR Master Mix (Thermo Fisher Scientific), 3 µl of diluted cDNA (1/15), 0.05 µl of uracil *N*-glycosylase (UNG) AmpErase (Thermo Fisher Scientific) and 1 µl of forward and reverse primers (300 nM, Table 2). Amplifications were performed in triplicate using an ABI 7500 Fast Real-Time PCR System (PE Applied Biosystems, Foster City, CA, USA) with the following cycling conditions: 2 min at 50 °C for AmpErase activation and 10 min at 95 °C for denaturation followed by 40 cycles of 15 s at 95 °C and 45 s at 60 °C. Melting curve analysis was carried out to ensure reaction specificity and search for primer-dimers artifacts. To obtain the relative mRNA quantity units, a standard curve was established in duplicate in each 96-well plate using serial dilutions of cDNA pools^36,37^. The relative quantification values were calculated by normalizing the relative quantity units of selected genes to those of the peptidylpropyl isomerase A (*PPIA*) gene that was used as a reference gene. This reference gene was not affected by body composition (fat *vs* lean) or the nutritional status (fasting *vs* feeding) of pigs, as determined with the NormFinder algorithm^38^ from Excel-Tools-Add-ins. Mean values from triplicates were used for statistical analyses.

**Table 2:** Primer sequences used for real-time PCR amplifications of studied genes.

Genes	Primer sequences (5′–3′)	GenBank accession no.	Product size (bp)	Amplification efficiency (%)
**Studied genes**				
*ACACA*	(F)CCGTAGAAATCAAATTCCGCAG (R)CCTTCAGCTTGCTCTCCAG	NM_001114269	141	98.8
*ACLY*	(F)TCACAACACCATCATCTGCG (R)CTTACTGAACATCTTGGCTGC	NM_001257276	124	101.3
*ADIPOQ*	(F)ATGATGTCACCACTGGCAAATTC (R)GACCGTGACGTGGAAGGAGA	EF601160	71	108.7
*ChREBP (MLXIPL)*	(F)ATGTTCGATGACTATGTCCGG (R)ACACCATCCCATTGAAGGAC	XM_013995540	103	100.0
*FASN*	(F)CTCAACTTCCGAGACGTCATG (R)ACCATTCCCATCACGCG	NM_001099930	124	102.0
*GAPDH*	(F)CCCCAACGTGTCGGTTGT (R)CTCGGACGCCTGCTTCAC	NM_001206359	91	95.7
*GCKR*	(F)TTCCCATTTCACCTTCTCCC (R)TTCTCTTTCACCTGCTCCAC	XM_013987930	141	98.1
*G6PD*	(F)AGATGATGACCAAGAAGCCC (R)GCAGAAGACGTCCAGGATG	XM_021080744	131	103.1
*LEP*	(F)GGCCCTATCTGTCCTACGTTGA (R)CTTGATGAGGGTTTTGGTGTCAT	NM_213840	71	94.5
*PPAR-γ*	(F)CCTTTGGTGACTTTATGGAGC (R)TCGATGGGCTTCACATTCAG	NM_214379	145	102.2
*SCD1*	CGGATATCGCCCTTATGACAAG CTCGCTGGCAGAATAGTCATAG	NM_213781	124	106.1
*SDHA*	GATTTGCGAACGGAACCATAAG GCTGCAAGTCTCCGTAGAG	XM_021076930	144	101.0
*SREBP-1c (SREBF1)*	(F)GCTTCCAGAGGGACCTGAG (R)CTCAGACTGCGGTCCAG	NM_214157	132	93.7
*SREBP-1a*	(F)CTGCTGACCGACATCGAA (R)GGAGCTGGCATCAGGAC	NM_214157	129	98.6
**Reference gene**				
*PPIA*	(F)GGTCCTGGCATCTTGTCCAT (R)TCATGCCCTCTTTCACTTTGC	NM_214353	71	97.4

*ACACA* acetyl CoA carboxylase alpha, *ACLY* ATP citrate lyase, *ADIPOQ* adiponectin, *ChREBP (MLXIPL)* MLX interacting protein like, *FASN* fatty acid synthase, *GAPDH* glyceraldéhyde-3-phosphate dehydrogenase, *GCKR* glucokinase regulator, *G6PD* glucose-6-phosphate dehydrogenase, *LEP* leptin, *PPAR-γ* peroxisome proliferator activated receptor gamma, *PPIA* peptidylpropyl isomerase A, *SCD1* stearoyl-CoA desaturase, *SDHA* succinate dehydrogenase complex flavoprotein subunit A, *SREBP-1c (SREBF1)* sterol regulatory element binding transcription.

### Statistical analysis

All the statistical analyses were performed using SAS software (version 9.4; SAS Institute Inc., Cary, NC, USA). To determine the relationship between insulin sensitivity and body composition, Spearman’s correlation analyses were performed between the insulin sensitivity indexes obtained during the OGTT with body lipid and protein percentage using the CORR procedure. Linear regression models were performed with the REG procedure to quantify the variation in body composition explained by insulin sensitivity. The dependent variables (body lipid or protein percentage) were regressed independently against each of the insulin-related index: insulin sensitivity (QUICKY, MI, and HOMA-IR) and insulin secretion indexes (HOMA-%B and oDI_cpep_).

The relative gene expression of each gene of fat and lean pigs studied was compared through a completely randomized design according to a 2 × 2 factorial arrangement with nutritional state (fasting or feeding) and type of pig (lean or fat) as the main factors, while the insulin-related indexes and de novo lipogenesis were compared through a completely randomized design with the type of pig as a fixed factor using the MIXED procedure. A multivariate Principal Component Analysis (PCA) was performed to assess the relationship among the quantitative variables (insulin sensitivity indexes, relative mRNA abundance and de novo lipogenesis). Raw data for this analysis were previously standardized using the Proc STANDARD of SAS.

### Ethics approval

All measurements and observations on animals were performed according to a recommended code of practice (Canada, 2012) and the guidelines of the Canadian Council on Animal Care (CCAC, 2009). The animal trial was approved by the Ethical and Animal Welfare Committee of the Sherbrooke Research and Development Centre, Agriculture and Agri-Food Canada (Sherbrooke, QC, Canada).

## Results

Four pigs from the original group of 30 did not finish consuming the oral glucose and 2 pigs lost their catheter during the experiment, thus data from those six pigs were not used in the current analysis. The total body lipids and total body protein proportions were on average 19.8 ± 1.5% and 16.3 ± 0.3%, respectively. The basal glucose and insulin plasma concentrations averaged 4.2 ± 0.2 mmol/l and 9.8 ± 4.7 µU/ml, respectively (Table 3). However, the CV among the pigs for basal insulin (CV = 48.0%) and AUC insulin (CV = 27.9%) were considerably higher than those obtained for basal glucose (CV = 5.3%) and AUC glucose (CV = 5.7%). The delta glucose and insulin concentrations, which indicate the increase in concentrations of glucose and insulin from basal concentrations to peak were on average 2.3 ± 0.9 mmol/l and 109.8 ± 43.5 µU/ml respectively. The delta glucose and insulin were also variable among pigs (CV = 40.6% vs. 39.6%, respectively). The basal plasma concentration of C-peptide averaged 97.5 ± 47.2 pmol/l, and, similar to plasma insulin, C-peptide basal concentration and AUC C-peptide were highly variable among pigs (CV = 42.0% and 27.8%, respectively).

**Table 3:** Descriptive statistics of the body composition, plasmatic glucose, plasmatic C-peptide, plasmatic insulin and insulin sensitivity indexes of pigs (n = 24) participating in the oral glucose tolerance test (trial 1).

Variables	Mean	SD	Minimum	Maximum
**Body conditions**				
Body weight, kg	94.8	3.4	88.3	101.2
Body lipids, %	19.8	1.503	16.2	22.32
Body protein, %	16.3	0.323	15.75	17.05
**Glucose, C-peptide and insulin concentrations**				
Basal glucose, mmol/l	4.2	0.2216	3.9	4.7
Basal C-peptide pmol/l	97.5	47.2	30.2	220.2
Basal insulin, uU/ml	9.8	4.7	3.6	19.4
Delta glucose, mmol/l	2.3	0.923	1.1	5.1
Delta insulin, uU/ml	109.8	43.49	50.4	204.1
AUC^1^ glucose, mmol × min/l	1485	84	1338	1690
AUC insulin, uU × min/ml	6220	1733	3025	9269
AUC C-peptide, pmol × min/l	50,505	14,049	29,409	77,917
**Insulin sensitivity indexes**				
QUICKI	0.36	0.03	0.31	0.41
Matsuda index (MI)	6.3	2.4	2.9	13.2
HOMA-IR	1.2	0.6	0.5	2.4
HOMA-%B	88.8	40.6	33.7	184.8
oDI_cpep_	539.4	157.1	254.1	853.6
C-peptide: insulin ratio	1.36	0.13	1.14	1.62

*AUC* Area under the curve, *QUICKI* quantitative insulin sensitivity check index, *oDI*_*cpep*_ oral disposition index, *HOMA-IR* homeostasis model assessment for estimating insulin resistance, *HOMA-%B* homeostasis model assessment for estimating β-cell function.

^1^Area under the curve of plasmatic glucose or insulin concentrations obtained from time 0 to 360 during the oral glucose tolerance test (OGTT).

### Insulin sensitivity from the oral glucose tolerance test and body composition

**Figure 1:**
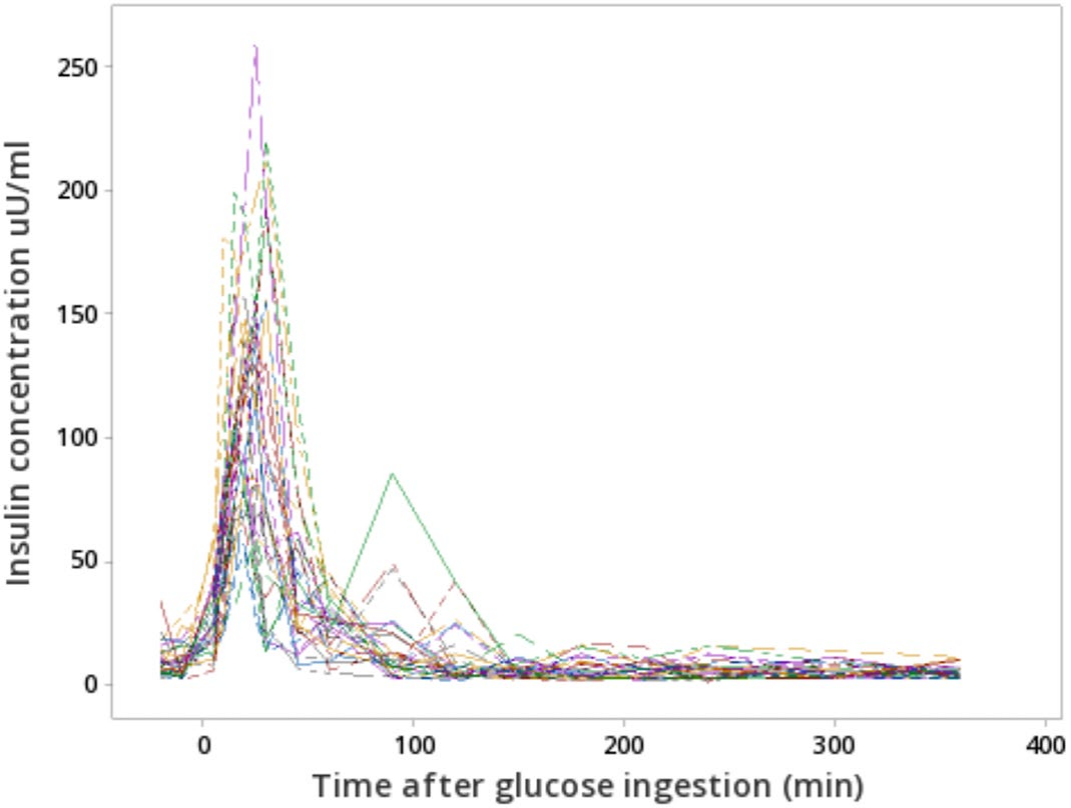
Plasmatic insulin concentration of 95 kg pigs after ingestion of a same dose of oral glucose (1.75 g/kg of BW).

**Figure 2:**
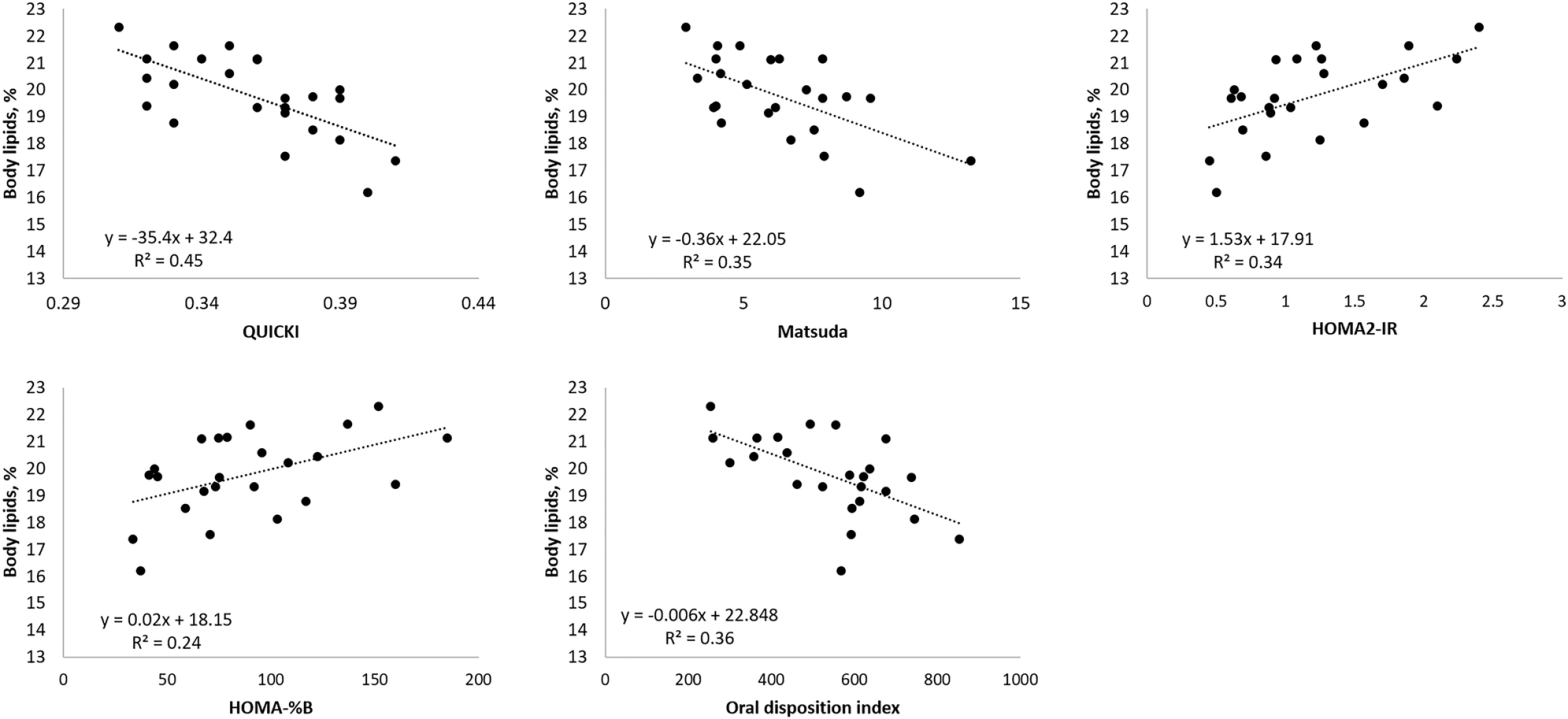
Linear regression among insulin sensitivity indexes and total body lipids (trial 1; n = 24). Only significant regressions with body lipids are presented in the figure (*P* < 0.05). *QUICKI* quantitative insulin sensitivity check, *oDI*_*cpep*_ oral disposition index, *HOMA-IR* homeostasis model assessment for estimating insulin resistance, *HOMA-%B* homeostasis model assessment for estimating β-cell function.

As was the case with insulin response (Fig. 1), most of the insulin-related indexes (MI, HOMA-%B, HOMA-IR and oDI_cpep_) were highly variable among pigs (Table 3). Moderate to strong correlations were found between whole body lipids percentage, whole body protein percentage and insulin sensitivity/secretion indexes (± 0.5 ≤ r ≤  ± 0.7; *P* < 0.05; Table 4) except for C-peptide: insulin ratio which indicates hepatic extraction and clearance of insulin. The regression analysis (Fig. 2) indicated that body lipid content linearly decreased as insulin sensitivity increased as estimated by the QUICKI and MI (β =  − 35.4 and β =  − 0.36; *P* < 0.01, respectively) indexes, whereas body protein content linearly increased with improved insulin sensitivity (Table 4). In addition, HOMA-IR linearly increased as the percentage of body lipids went up (β = 1.5; *P* < 0.01). Insulin secretion during the basal state (HOMA-%B) linearly increased as the percentage of body lipids went up (β = 0.02; *P* < 0.05). The opposite relationship was found with body protein content (Table 4). When glucose concentration increased after oral glucose ingestion (absorptive state), β-cell function, measured by oDI_cpep_ linearly decreased as body lipids content increased (β =  − 0.006; *P* < 0.01).

**Table 4:** Correlations between body composition and insulin sensitivity and secretion indexes estimated from the oral glucose tolerance test (trial 1; n = 24).

	Total body lipids		Total body protein	
	r	*P*	r	*P*
**Insulin sensitivity**				
QUICKI	− 0.67	< 0.001	0.66	< 0.001
Matsuda index (MI)	− 0.59	< 0.01	0.60	< 0.01
HOMA-IR	0.58	< 0.01	− 0.59	< 0.01
C-peptide:insulin ratio^1^	− 0.31	> 0.05	0.29	> 0.05
**Insulin secretion**				
HOMA-%B	0.45	< 0.05	− 0.48	< 0.05
oDI_cpep_	− 0.60	< 0.01	0.58	< 0.01

*QUICKI* quantitative insulin sensitivity check index, *oDI*_*cpep*_ oral disposition index, *HOMA-IR* homeostasis model assessment for estimating insulin resistance, *HOMA-%B* homeostasis model assessment for estimating β-cell function.

^1^AUC C-peptide/AUC insulin.

### Gene expression and de novo lipogenesis in the adipose tissue of fat and lean pigs

**Figure 3:**
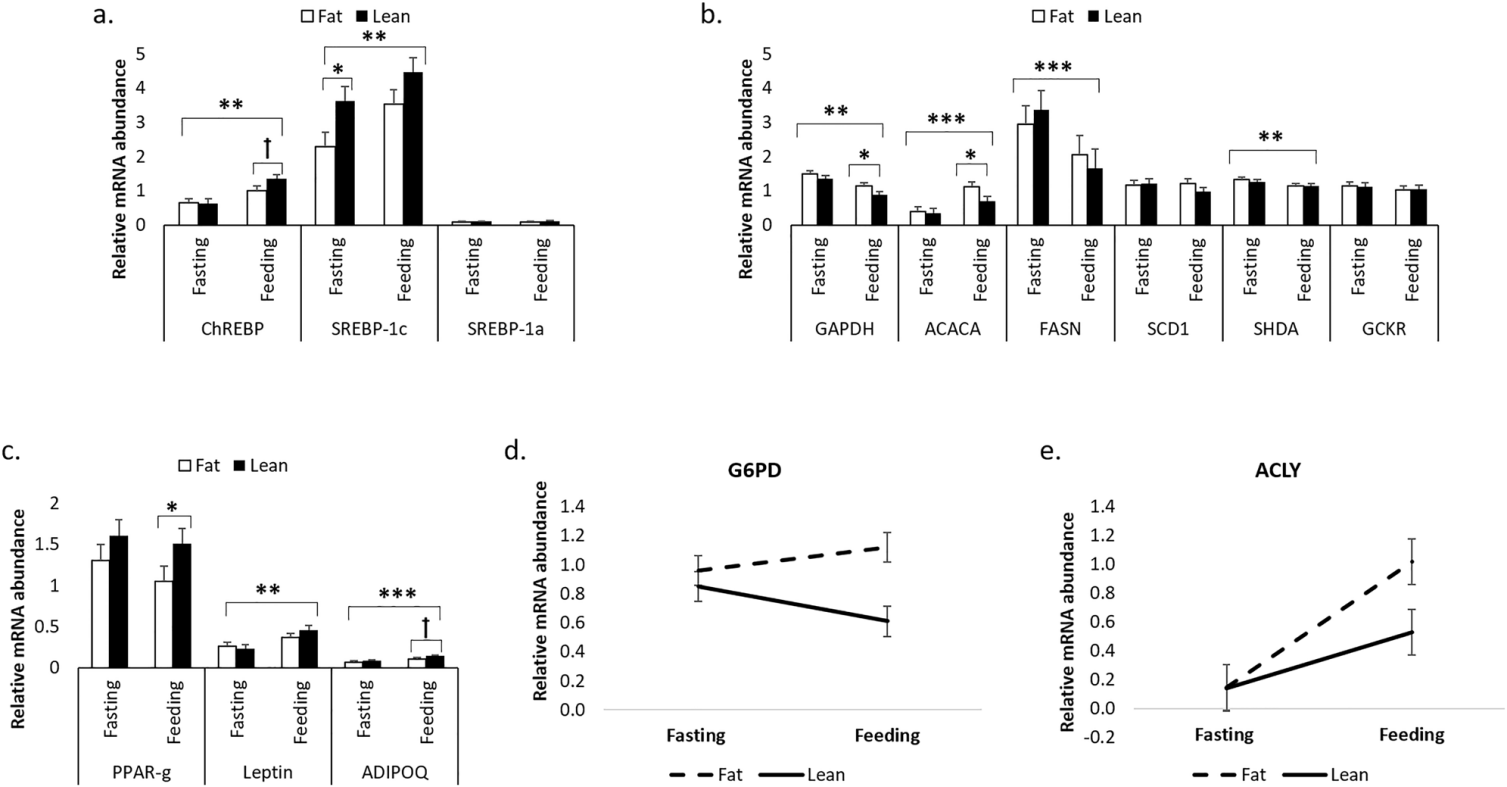
Relative mRNA abundance of transcription factors (**a**), enzymes implicated in lipid metabolism (**b**) and genes associated with insulin sensitivity (**c**) in fat and lean pigs during fasting and feeding (trial 2). Interactions between nutritional state and type of pigs for *G6PD* and *ACLY* are illustrated in (**d,e**). Error bars represent SE. *,**,*** and ꝉ stands for *P* < 0.05, *P* < 0.01, *P* < 0.001 and *P* < 0.10, respectively (n = 14).

The body lipid content of lean and fat pigs was on average 17.4 ± 0.9% and 22.0 ± 1.0%, respectively. Insulin sensitivity and β-cell function were higher in lean pigs compared with fat pigs (QUICKI = 0.38 vs. 0.34, and oDI_cpep_ = 674.4 vs. 421.3; *P* < 0.05). Some of the studied genes mRNA abundance was up-regulated (*ChREBP*, *SREBP-1c*, *ACACA*, *LEP*, ADIPOQ) or down-regulated (*GAPDH*, *FASN*, *SHDA*) after feeding (*P* < 0.01; Fig. 2a–c). During the fasting state, only the relative mRNA abundance of *SREBP-1c* was higher in lean pigs than in fat pigs (3.62 vs. 2.28; *P* < 0.05; Fig. 3a), whereas the relative mRNA abundance of the other genes remained similar between fat and lean pigs. In the feeding state, *GAPDH* and *ACACA* had lower relative mRNA abundance in lean pigs than fat pigs (*P* < 0.05; Fig. 3b), while that of the protein regulator *PPAR-γ* was higher for the lean pigs (1.06 vs. 1.51; *P* < 0.05; Fig. 3c). In addition, there was a trend for higher relative mRNA abundance for the transcription factor *ChREBP* and the adipokine adiponectin (*ADIPOQ*) in lean pigs than in fat pigs (Fig. 3a,b).

**Figure 4:**
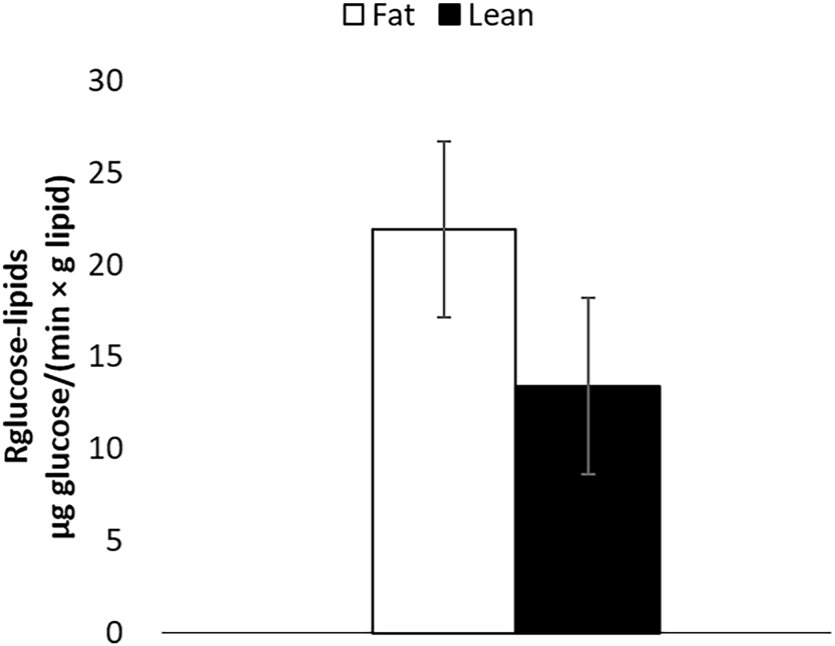
Estimations of the rate of glucose incorporation into lipids (R_glucose-lipids_) of fat and lean pigs at 4 h after a bolus injection of [U-^13^C]glucose (trial 2). Error bars represent SE (n = 14).

An interaction between nutritional state and type of pigs was observed for *G6PD* (*P* < 0.05) and *ACYL* (*P* < 0.05; Fig. 3d,e), indicating that the differences in mRNA abundance are more important during the feeding period, when compared with fasting. The R_glucose-lipids_ at four hours after [U-^13^C]glucose injection of fat and lean pigs was on average 21.9 ± 13.5 µg glucose/(min × g of lipids) and 13.4 ± 8.9 µg glucose/(min × g of lipids), respectively. However, despite the observed numerical difference between fat and lean pigs, R_glucose-lipids_ was not significantly different between the two groups (*P* = 0.20; Fig. 4). Additionally, correlations of R_glucose-lipids_ with QUICKI (r =  − 0.58), HOMA-%B (r = 0.62) and HOMA-IR (r = 0.62) were significant (*P* < 0.05), but not with whole-body lipid content (r = 0.39; *P* = 0.18).

#### Principal component analysis of insulin-related indexes, body composition variables and gene expression

**Figure 5:**
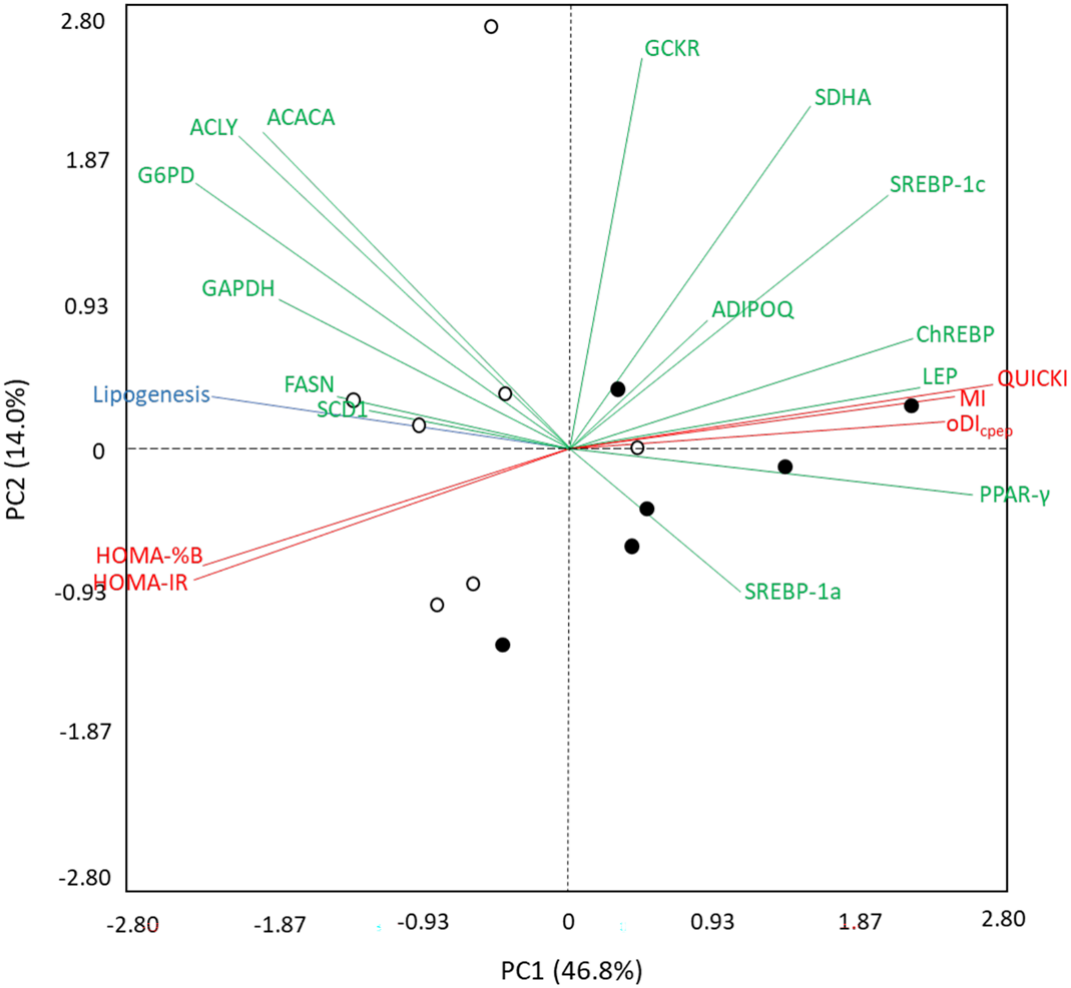
Principal components analysis (PCA) constructed with the gene expression (green), insulin sensitivity/secretion indexes (red) and stable isotopes (blue) variables of fat (open circle) and lean (filled circle) pigs. *PC* principal component, *ACACA* acetyl CoA carboxylase alpha, *ACLY* ATP citrate lyase, *ADIPOQ* adiponectin, *ChREBP* MLX interacting protein like, *FASN* fatty acid synthase, *GAPDH* glyceraldehyde-3-phosphate dehydrogenase, *GCKR* glucokinase regulator, *G6PD* glucose-6-phosphate dehydrogenase, *LEP* leptin, *PPAR-γ* peroxisome proliferator activated receptor gamma, *SCD1* stearoyl-CoA desaturase, *SDHA* succinate dehydrogenase complex flavoprotein subunit A, *SREBP* sterol regulatory element binding transcription *QUICKI* quantitative insulin sensitivity check, *MI* Matsuda index, *HOMA-IR* insulin resistance, *HOMA-%B* β-cell function, *oDI*_*cpep*_ oral disposition index (n = 13).

The first two components of the PCA accounted for 60.8% of the total variance (PC1 = 46.8%; PC2 = 14.0%), and fat and lean pigs were clustered in two separated groups (Fig. 5). The PC1 was mostly defined by insulin sensitivity indexes (QUICKI and MI) and genes associated with insulin sensitivity (*PPAR-γ* and *Lep*), while the PC2 was mostly defined by the relative mRNA abundance of genes that participate in the de novo lipogenesis pathway (*GP6D*, *SDHA*, *GCKR*, *ACACA* and *ACLY*). PC1 had positive strong correlations (r > 0.7; *P* < 0.01) with QUICKI, MI, oDI_cpep_, *PPAR-γ, Lep* and total body protein, but was negatively correlated (r <  − 0.7; *P* < 0.01) with HOMA2-IR, HOMA-%B, *G6PD*, de novo lipogenesis and total body lipids. The PC2 had strong positive correlations only with *SDHA* and *GCKR* (r > 0.7; *P* < 0.01) and moderate positive correlations (r > 0.6*; P* < 0.05) with *ACACA and ACLY.* It can be observed that de novo lipogenesis was negatively associated with *PPAR-γ, Lep*, QUICKI, MI and oDI_cpep_, while these five variables were positively associated with each other.

## Discussion

The first objective of this study was to investigate whether variations in the observed body composition are related to differences in insulin sensitivity among pigs. Results from this study clearly showed that pigs raised in the same conditions and having the same genetic background and same feed intake can respond differently to a given diet. This was indeed demonstrated in the first trial where important variations in basal plasma insulin concentrations and AUC_0–360_ response to OGTT were observed among pigs, while plasmatic glucose measurements remained relatively constant. The observed variations in plasmatic insulin among pigs could be explained, at least in part, by variability in insulin sensitivity (Supplementary Table S1). The basal concentrations of glucose and insulin obtained in trials 1 and 2 are similar to those previously reported in Duroc boars of 145.8 ± 16.8 kg BW^26^. Significant individual variations in insulin response and sensitivity were reported in healthy humans^39^. Correlations of insulin sensitivity, assessed with OGTT indexes, and backfat thickness were previously reported in pigs on different fish oil diets^26^, and are in agreement with results from the current study. It is generally accepted that obesity and body fat distribution are closely associated with insulin resistance, and that this association may explain, at least in part, the heterogeneity observed in insulin sensitivity in healthy human populations^12,40^. Study results also showed that the β-cell function in its basal state (HOMA-%B) increases when body lipids increase, which may be explained by a compensatory insulin secretion from pancreatic β-cells in response to the lower insulin sensitivity of fatter pigs^41^. In addition, the significant correlation of MI and C-peptide/glucose (r =  − 0.72; *P* < 0.001) suggest that fat pigs with lower insulin sensitivity increased insulin secretion during the OGTT. However, during the glucose challenge, the observed linear decrease of oDI_cpep_ when body lipids increase rather indicates there is insufficient compensation at the pancreas for losses in whole-body insulin sensitivity. An important part of the between-animal variations in total body lipids and total body protein content was associated with the insulin sensitivity (QUICKI, MI and HOMA-IR) and insulin secretion (oDI_cpep_) indexes, as demonstrated by the regression analyses.

The second objective of this study was to determine whether the expression of genes associated with insulin sensitivity and lipid metabolism differ between fat and lean pigs under similar nutrient intakes. The differences between fat and lean pigs in gene expression of enzymes participating in lipid synthesis (*GAPDH*, *ACACA*, *G6PD*, *ACLY*) were observed during the feeding period but not during fasting. The feeding period is known to raise insulin concentrations and to stimulate lipid synthesis^42^. Therefore, differences among fat and lean pigs observed during the feeding period may be associated, at least in part, with metabolic pathways in which insulin signalling is involved. For example, the higher secretion of insulin in fat pigs with low insulin sensitivity might be associated with their greater expression of *G6PD* and *ACLY*, observed after the meal but not during fasting*,* as those genes are particularly stimulated by insulin during feeding^43,44^.

In this study, fat pigs with low insulin sensitivity had higher *G6PD* mRNA abundance and lower *PPAR-γ* abundance when compared with the lean pigs. The overexpression of *G6PD* has been associated with lipid dysregulation and low insulin sensitivity in obese mouse models^11^. In fact, this gene can negatively affect insulin sensitivity by two ways. First and foremost, its overexpression in the adipose tissue leads to an increase in cellular NADPH level, stimulating the lipogenic activity in adipocytes^11^. This can lead to lipid accumulation, which positively correlates with losses in insulin sensitivity, independently of the animal’s obesity status^45^. Secondly, the *G6PD* overexpression alters the expression of adipokines, thus increasing the expression of resistin while decreasing that of adiponectin^11^. This is in agreement with the results of the current study where the increase in *G6PD* mRNA abundance in fat pigs is associated with a decrease in *ADIPOQ* transcript abundance (tendency). Adiponectin acts as a mediator of insulin activity in peripheral tissues and protects non-adipose tissues against an excessive lipid overload while maintaining normal organ function^46^. The lower mRNA abundance of *PPAR-γ* in the sc adipose tissue of fat pigs when compared with the lean pigs (feeding state) was unexpected since this nuclear hormone receptor has been identified as a master regulator of adipocyte differentiation and an essential mediator of whole-body insulin sensitivity^47^. Conflicting observations regarding the expression of *PPAR-γ* in the adipose tissue of obese patients from different studies were recently reported by Torres et al.^48^, with some studies showing increased expression of *PPAR-γ* in obese subjects *vs* controls, whereas others showed decreased expression or no differences. As suggested by these authors, discrepancies among studies may be explained by differences in gender, fat depots and insulin sensitivity. Benitez et al.^49^ also demonstrated that the timing when adipose tissue samples are collected is of importance when measuring *PPAR-γ* expression. Indeed, the *PPAR-γ* expression in adipose tissue was higher in growing (44 kg BW) then in the finishing (100 kg BW) pigs^49^, in agreement with a more intense pre-adipocytes differentiation expected at younger age. With respect to insulin sensitivity, it is worth nothing that besides the *PPAR-γ* receptor transcript being more abundant in lean than fat pigs, our study also showed a positive correlation between the expression of *PPAR-γ* and the QUICKI index assessing insulin sensitivity (r = 0.71; *P* < 0.01). The role of *PPAR-γ* in adipocytes differentiation is well established, but additional functions are starting to emerge. For example, it has been documented that adipose *PPAR-γ* guarantees the balance and adequate production and secretion of adiponectin and leptin, also known to mediate insulin action in peripheral tissues^46^.

In fat pigs, the observed increase in the mRNA abundance of enzymes participating in de novo lipogenesis (ex. *ACACA*, *ACLY*, *G6PD*, *GAPDH*) can be explained, at least in part, by the increase in insulin secretion observed in fat pigs having lower insulin sensitivity. In fact, it was previously demonstrated that insulin can stimulate the expression of the lipogenic enzymes *ACACA* and *ACLY*^44^. In addition, an indirect effect of insulin on the expression of lipogenic enzymes is also possible because de novo lipogenesis in the liver can be upregulated at the transcriptional level by the overexpression of *SREBP-1c* and *ChREBP* genes, two transcription factors known to be stimulated by insulin and carbohydrates, respectively^50^. However, the lack of differences in the mRNA abundance of *SREBP-1c* between fat and lean pigs during feeding indicates that the activation of *ACACA* and *ACLY* transcription would be subjected to a mechanism that is independent of *SREBP1-c* transcription. In agreement with our results, previous studies have demonstrated that the mRNA expression of *SREBP1-c* has no effect on the lipogenic genes in adipocytes in contrast to hepatocytes^51,52^. Nevertheless, we cannot rule out the possibility that the activation of SREBP-1c by the SREBP cleavage-activating protein (SCAP) and two proteases (S1P and S2P), followed by its translocation into the nucleus may, in turn, contribute to the observed increase in the expression of lipogenic enzymes^53^. Moreover, it is well known that changes in mRNA abundance do not always reflect differences in protein expression or activities. For example, some enzymes involved in de novo lipogenesis can undergo post-translational modifications in response to feeding. This is the case for ACLY, which demonstrated greater activity following its phosphorylation^54^. On the other hand, ACACA and FASN activities were inhibited by phosphorylation^55,56^. Therefore, the lack of effect between fat and lean pigs at the transcriptional level for lipogenic genes such as *SREBP-1c, FASN* and *SCD1* does not rule out the possibility of post-translational modifications and activation that may affect activities and, in turn, the de novo lipogenesis.

Fasting is known to reduce lipogenesis and lipogenic gene expression in adipose tissue^57^. In agreement, the relative mRNA abundance of *ChREBP*, *SREBP-1C*, *ACACA*, *LEP* and *ADIPOQ* were all down regulated in fasting than in the feeding state. The down regulation of *GAPDH*, *FASN* and *SHDA* expression in the feeding state was unexpected (current study), as was the lack of effects of fasting and refeeding on the expression of *ACACA*, *FASN* and *LEP* in the ham adipose tissue of Iberian fatty pigs^49^. The different breeds used and the location of adipose tissue sampling may account for some of the observed discrepancies, but additional experiments are needed to identify the mechanisms leading to a reduced expression of lipogenic genes such as *FASN* in the feeding state. Only the mRNA abundance of *SREBP-1C* was significantly different between lean and fat pigs in the fasting state. The unexpected reduction of *SREBP-1C* mRNA abundance observed in fat *vs* lean pigs was also observed in the sc adipose tissue of obese *vs* normal weight or lean human subjects^58,59^. Moreover, Kolehmainen et al.^58^ suggested that the reduction in *SREBP-1C* expression found in obese subjects may be a consequence of an insulin resistance state that commonly develop with obesity. Interestingly, fat pigs from the present study had reduced insulin sensitivity when compared with the lean ones. The effect of insulin on *SREBP-1C* expression may therefore be attenuated due to the observed reduction of insulin sensitivity in fat pigs (current study).

The average values of R_glucose-lipids_ observed in this study in fat and lean pigs are in agreement with previous studies using radioactive glucose to estimate lipogenesis^8,32^. Even if fat pigs, in average, had an higher rate of lipogenesis by 65%, when compared with lean pigs, the large variations in R_glucose-lipid_ values between animals within each group of pigs can account for the lack of statistical difference (fat pigs: CV = 62%; lean pigs: 67%). Finally, correlations among de novo lipogenesis, body lipids content and insulin-related indexes indicated that lipogenesis is more likely associated with low insulin sensitivity and insulin secretion than body lipid content.

Insulin, along with branch-chain amino acids, also regulates protein synthesis. In young, healthy lean humans, skeletal muscle insulin resistant seems to be the first sign of onset type 2 diabetes^60^. Although the current study focused on the relationship between whole-body lipid mass and insulin sensitivity, the importance of the skeletal muscle on the whole-body insulin sensitivity cannot be ignored^61^. Based on the important correlations among whole body protein and insulin sensitive found in our study, one might hypothesize that pigs with high protein deposition were those with improved insulin sensitivity. In fact, increases in adipose tissue is known to increase inflammation and lipotoxicity, whose will negatively impact insulin sensitivity decreasing protein synthesis and/or inhibit protein degradation in muscle^60^. Granting that several theories surround this subject, it seems that a link between hyperlipidemia-induced reactive oxygen species production in the skeletal muscle and insulin resistance can be stablished^62^. Such metabolic change incurs greater lipid availability and usage potentially inducing oxidative stress that inhibits branch chain amino acids catabolic enzymes^60^.

The PCA was performed to study the associations between the observed metabolic responses in plasma (e.g. insulin sensitivity indexes and secretion), gene expression of selected genes, de novo fatty acid synthesis and the body composition of finishing pigs. This analysis indicated that PC1 accounted for most of the observed variations (47%) compared with PC2, which accounted for 14%. The clustering of fat and lean pigs was mainly determined by the insulin sensitivity index variables and the mRNA abundance of some genes that defined PC1, however, de novo lipogenesis also played a major role. Lean pigs were associated with insulin sensitivity indexes (QUICKI, MI, and oDI_cpep_) and mRNA expression of *PPAR-γ* and *LEP*. The association among these variables indicates that the improved insulin sensitivity observed in lean pigs is positively related to the mRNA expression of *PPAR-γ* and *LEP.* As previously discussed, the expression of these genes is important to mediate insulin sensitivity in the whole body. Leptin is mainly produced in the adipose tissue^63^ and its secretion increases energy expenditure, reduces body fat^64^ and helps in maintaining adequate whole body insulin sensitivity^65^. On the other hand, fat pigs were associated with reduced insulin sensitivity (HOMA-IR), increased insulin secretion (HOMA-%B) and increased de novo lipogenesis. The positive relationship among these variables suggests that the increase in de novo lipogenesis observed in fat pigs may be associated with higher insulin secretion since insulin acts as a lipogenic hormone in the adipose tissue^6^. However, in a scenario of decreased whole-body insulin sensitivity, adipose tissue is supposed to be less responsive to insulin and should not cause increased lipogenesis, which is opposite to our results. In this case, two scenarios can be proposed to support our data. One is that an impaired suppression of endogenous glucose production by insulin happens in the liver but not in the adipose tissue or muscle^66^, then the pancreas secrets more insulin to compensate the hepatic insulin resistance, which increases adipose lipogenesis as the insulin anabolic effect on lipid synthesis remains unaffected in the adipose tissue. A second scenario suggests a *cis* selective insulin resistance where not all the insulin/akt-regulated process (glucose transport, protein synthesis, lipid synthesis, antilipolysis) are affected by insulin resistance^67^. For example, adipose tissue from mouse models show that insulin-mediated suppression of lipolysis is largely unaffected in insulin resistance despite impaired insulin-stimulated glucose transport, especially at higher doses of insulin^68^, therefore, the same scenario might be possible but for lipogenesis stimulated by insulin. Nevertheless, both scenarios must be validated in further experiments.

In addition, the negative association of *G6PD* expression with PC1 suggests that its negative effect on insulin sensitivity also affects fat pigs. Overall, the multivariate analysis indicates that variables related to insulin sensitivity show significant associations with dietary energy and nutrient utilization, as well as with body composition in pigs.

## Conclusion

Results from the present study clearly showed that insulin sensitivity is negatively correlated with total body lipids in pigs and that insulin sensitivity explained about 45% of the total body lipid and protein variations among pigs. When compared with lean pigs, pigs with elevated total body lipids had lower insulin sensitivity and higher gene expression of lipogenic enzymes (*ACACA* and *ACLY*). In addition, the higher abundance of *G6PD* transcripts along with the lower abundance of *PPAR-γ* may have contributed to lowering insulin sensitivity in fat pigs since these two genes are known to affect insulin sensitivity and to mediate insulin action in peripheral tissues. Observed relationship among variables (PCA analysis) suggests that fat pigs increase insulin secretion as a compensatory mechanism for losses in insulin sensitivity, which is positively associated with de novo lipogenesis and the up-regulation of two lipogenic genes (*ACACA* and *ACLY*). Overall, this study demonstrates that insulin sensitivity is an important factor determining the utilization of dietary energy and nutrients with implications for body composition in finishing pigs.

## Supplementary Information

Supplementary Table S1.

## Data Availability

The data that support the findings of this study are available from Her Majesty the Queen in Right of Canada as represented by the Minister of Agriculture and Agri-Food, but restrictions apply to the availability of these data, which were used under license for the current study, and so are not publicly available. Data are however available from the corresponding author upon reasonable request and with permission of Her Majesty the Queen in Right of Canada as represented by the Minister of Agriculture and Agri-Food.
