# Supplementary material for: Insulin sensitivity is associated with the observed variation of de novo lipid synthesis and body composition in finishing pigs
Source: Sci Rep. 2022 Aug 26;12:14586. doi: 10.1038/s41598-022-18799-0 (PMC9418310; doi:10.1038/s41598-022-18799-0)
Supplement: Supplementary file 1 — Supplementary Table S1. [file 41598_2022_18799_MOESM1_ESM.docx]

**Supplementary Material**

**Insulin sensitivity is associated with body composition, *de novo* lipid synthesis and adipose tissue’s gene expression of finishing pigs**

*Hector Hernando Salgado*^1,2^*, Candido Pomar*^1,2^, *Marie France Palin*^1^*, Hélène Lapierre*^1^*, Marie-Pierre Létourneau-Montminy^2^, John P. Cant^3^, Aline Remus^1,*^*

***Table s1.*** *Correlations among* *insulin sensitivity indexes (QUICKI and Matsuda) with parameters of plasmatic insulin and glucose kinetics during an oral glucose tolerance test (1.75 g of glucose/kg of BW) in 95 kg pigs (n = 24).*

|  | QUICKI | |  | Matsuda | |
| --- | --- | --- | --- | --- | --- |
|  | r | *P-value* |  | r | *P-value* |
| Peak of insulin | -0.40 | 0.05 |  | -0.73 | 0.00 |
| Peak of glucose | -0.95 | 0.00 |  | -0.88 | 0.00 |
| AUC^1^ glucose | -0.03 | 0.90 |  | 0.05 | 0.82 |
| AUC insulin | -0.54 | 0.01 |  | -0.81 | 0.00 |

AUC = Area under the curve; QUICKI = Quantitative Insulin Sensitivity Check.

^1^ Area under the curve of plasmatic glucose or insulin concentrations obtained from time 0 to 360 during OGTT.
